# Extracellular Vesicles From *Schistosoma mansoni* Adult Worms Stimulate IL‐10 Release by B Cells

**DOI:** 10.1111/pim.70023

**Published:** 2025-09-07

**Authors:** Marije E. Kuipers, Arifa Ozir‐Fazalalikhan, Simone Haeberlein, Nicole N. Driessen, Clarize M. de Korne, Lynn Mes, Paul J. Hensbergen, Esther N. M. Nolte‐’t Hoen, Cornelis H. Hokke, Hermelijn H. Smits

**Affiliations:** ^1^ Department of Parasitology Leiden University Medical Center Leiden the Netherlands; ^2^ Department of Biomolecular Health Sciences, Faculty of Veterinary Medicine Utrecht University Utrecht the Netherlands; ^3^ Leiden University Center for Infectious Diseases Leiden University Medical Center Leiden the Netherlands; ^4^ Interventional Molecular Imaging Laboratory, Department of Radiology Leiden University Medical Center Leiden the Netherlands; ^5^ Center for Proteomics and Metabolomics Leiden University Medical Center Leiden the Netherlands

**Keywords:** adult worms, extracellular vesicles, IL‐10, proteomics, regulatory B cells, *Schistosoma mansoni*

## Abstract

Schistosome parasites are known to modulate host immune responses, which is achieved in part through the release of excretory/secretory (ES) products, including extracellular vesicles (EVs). During chronic schistosomiasis, increased regulatory responses are found, which include enhanced IL‐10 production by B (Breg) cells. ES products from schistosome eggs are able to induce IL‐10 production by B cells. However, since infection with male worms only (without egg production) also promotes IL‐10 producing B cells, we here studied the stimulatory effects of adult worm ES and EVs on murine and human B cells. Worm ES increased IL‐10 release by mouse splenic B cells; this activity was concentrated in defined size‐separated fractions of adult worm ES. Interestingly, mass spectrometry of the fractions that induced the highest IL‐10 response revealed an enrichment of EV‐associated proteins. Indeed, highly purified adult worm EVs could interact with mouse splenic B cells, visualised by binding of a schistosome‐specific tetraspanin (TSP2) targeting antibody. Furthermore, purified adult worm EVs induced IL‐10 release in both mouse splenic and human peripheral blood B cells, suggesting that adult worm EVs can play a role in immune regulatory processes within their host.

## 1 Introduction

*Schistosoma* spp. are endemic in tropical areas and globally affect over 250 million people [1]. These parasites are known to regulate host immune responses, enabling the adult worms to survive in their human host for many years, despite being exposed to the full army of immune defences present in the blood [2]. Enhanced knowledge of how schistosomes can avoid and attenuate immune responses contributes to our understanding of immunity against schistosomes and aids the development of vaccines. Furthermore, understanding parasite–host interactions can reveal new mechanisms to dampen immune responses, which we can implement in novel strategies to treat inflammatory disorders [3].

Schistosomes can modulate both innate and adaptive immune responses of their hosts. One crucial mechanism for increased survival of both the parasites and their host is the dampening of immune responses via stimulation of the regulatory arm of the immune system [4]. This regulatory arm consists of both innate (e.g., dendritic cells and macrophages) and adaptive (e.g., T and B cells) cells and involves the release of regulatory cytokines such as TGF‐β and IL‐10 by these cells. Stimulation of regulatory responses by helminths, including schistosomes, is often studied by exposing immune cells to soluble antigen extracts of the parasites, released excretory/secretory (ES) products and/or single antigen/ES components [5]. A previously overlooked component that is studied among the ES products is extracellular vesicles (EVs) [6]. EVs are released by cells of almost every organism and are generally 50–200 nm in size with a lipid‐bilayer membrane and diverse cargo (e.g., (glyco)proteins, lipids and RNAs) [7]. Although several studies have investigated the composition of schistosome‐derived EVs [8, 9, 10, 11, 12, 13, 14], information regarding their role in modulating the host immune system is limited [15, 16].

Regulatory B (Breg) cells are a collective name for a heterogenous group of B cells with anti‐inflammatory activity, which are relatively new to the framework of regulatory immune cells. They can suppress pro‐inflammatory responses via the release of anti‐inflammatory cytokines such as IL‐10, IL‐35 and/or TGF‐β, or via cell‐contact dependent mechanisms. Breg cells can drive effector T cell suppression and induce regulatory T cells, but they can also inhibit cytokine production by antigen‐presenting cells or drive T cell apoptosis [17]. IL‐10 producing Breg cells are induced during schistosome infections [18, 19]. Additionally, in vivo and in vitro studies with *S. mansoni* soluble egg antigens (SEA), without the context of a full infection, showed that SEA can induce IL‐10 production in both mouse and human B cells, respectively [20, 21]. However, during an *S. mansoni* infection, IL‐10 producing Breg cells are not exclusively induced by schistosome egg‐derived components. Intriguingly, infections with male worms only, and thus in the absence of eggs, could still protect mice against experimental anaphylaxis in a B cell‐ and IL‐10‐dependent manner [22]. So far, it is unknown whether ES products or other components of adult worms influenced B(reg) cell function in this model.

Hence, we investigated the effect of *S. mansoni* adult worm ES on mouse and human B cell cytokine release. Adult worm ES induced IL‐10 release by mouse splenic B cells. Interestingly, proteomics on IL‐10 inducing ES‐fractions, generated by size exclusion chromatography, revealed an enrichment in EV‐related proteins. Indeed, stimulation with highly purified EVs from adult worms could increase IL‐10 release by naïve mouse splenic B cells and by human peripheral blood B cells. This suggests that adult worm EVs harbour immune suppressive potential when exposed to B cells.

## 2 Methods

### 2.1 Ethical Statement

All animal experiments were in accordance with the Guide for the Care and Use of Laboratory Animals of the Institute for Laboratory Animal Research and have received approval from the university Ethical Review Board (Leiden University Medical Center, Leiden, Netherlands) which is registered with the number AVD1160020171067 (for hamsters) and AVD1160020173525 (for mice). Healthy volunteers provided informed consent to collect venous whole blood, which was approved by the Institutional Review Board of Leiden University Medical Centre.

### 2.2 Worm Culture and ES Collection

*S. mansoni* adult worms (mixed sex) were collected from perfused Syrian hamsters, washed multiple times and cultured ~10–20 worms/mL DMEM (high glucose with L‐glutamine, Lonza, Basel, Switzerland) supplemented with Antibiotic Antimycotic Solution (ABAM, Sigma‐Aldrich, St. Louis, MO, United States) and 10 mM HEPES for two days as previously described in detail [14]. Collected ES was centrifuged at 300 × *g* for 5 min to remove worms and debris and the supernatant was stored at −20°C until further use. Collected ES for subsequent EV isolation was centrifuged twice at 200 × *g* and twice at 500 × *g*, all for 10 min at 4°C. The final supernatant was stored at −80°C until further use. For the experiments with total ES and ES fractionation, worms were cultured for two days in M199 medium (Gibco, Thermo Fischer Scientific, Waltham, MA, USA) supplemented with 1.5 mM glutamine, 10 mM HEPES and ABAM. Generation of SEA from *S. mansoni* eggs has been described elsewhere [20]. Protein concentration of ES was determined by BCA (Pierce, Thermo Fisher Scientific).

### 2.3 FPLC Fractionation

Thawed worm ES from 310 to 502 mL was centrifuged at 1800 × *g* for 10 min (4°C) and the supernatant was concentrated in 10 kDa centrifugal filters (Amicon, Merck KGaA, Darmstadt, Germany) with three additional PBS washings till a volume of 1–2 mL. The concentrated ES was centrifuged twice in Eppendorf tubes at 17,000 × *g*. For each FPLC run, 0.8–1 mL of the final supernatant was applied to a Sephacryl S‐300 HR column (Pharmacia, Stockholm, Sweden) and eluted with PBS using an ÄKTA FPLC system (Pharmacia). Fractions of 1 mL were collected and stored at −20°C till further use. A chromatogram of a representative run can be found in Figure S1A. For the B cell stimulations, collected matching fractions from 3 runs were pooled and the protein concentration was determined by NanoDrop (Thermo Fischer Scientific).

### 2.4 SDS‐PAGE and Mass Spectrometry

Pooled FPLC fractions were mixed with non‐reducing sample buffer before loading 20 μL onto a 10% SDS‐PAGE gel followed by Coomassie staining or blotting on a PVDF membrane. The membrane was further processed for ConA (Concanavalin A) lectin staining (biotinylated, Vector laboratories, Burlingame, CA, United States) as previously described [14]. The gel was kept in MilliQ and cut into selected pieces (Figure S1B). Proteins in sliced gel bands were digested with trypsin (12.5 ng/μl in 25 mM NH_4_HCO_3_, sequencing‐grade modified trypsin; Promega, Madison, WI, USA) after reduction and alkylation with dithiothreitol (10 mM) and iodoacetamide (55 mM), respectively. Tryptic peptides were then analysed by LC–MS/MS using an Ultimate 3000 RSLCnano system (Thermo Fisher Scientific) coupled to an ion trap mass spectrometer (amaZon ETD [Bruker, Billerica, MA, USA]). The raw MS proteomics data have been deposited to the ProteomeXchange Consortium via the PRIDE partner repository with the dataset identifier PXD044023 [23].

For peptide identification, mass spectrometry data were converted to Mascot Generic Files (MGF) and searched against the Uniprot *Schistosoma mansoni* database (Downloaded 19 November 2020, 15,910 protein entries) with the Mascot search algorithm (Mascot 2.2.07, Matrix Science, London, UK). A MS tolerance of 0.3 Da and a MS/MS tolerance of 0.5 Da were used. Trypsin was selected for enzyme specificity and up to two missed cleavage sites were allowed. Carbamidomethyl cysteine was selected as a fixed modification, and oxidation of methionine and acetylation at the protein N‐terminus as variable modifications. All searches and subsequent data analysis, including Percolator [24], were performed using Proteome Discoverer 2.5 (Thermo Fischer Scientific). Peptide‐spectrum matches were adjusted to a 1% FDR. Proteins with only 1 detected peptide were excluded from further analysis. For comparing our proteome to published EV proteomes, we combined three publications on *S. mansoni* adult worm EVs [9, 11, 12] and one publication on *S. mansoni* schistosomula EVs [8]. Since previously published proteomes were annotated with smp accession numbers, these entries were IDmapped to Uniprot entries via the Uniprot website (http://uniprot.org, consulted in Augustus 2022). Sixty entries could not be mapped (see Supporting Information) and were therefore not included in the comparison.

### 2.5 ES Preparation and EV Isolation

**FIGURE 1:**
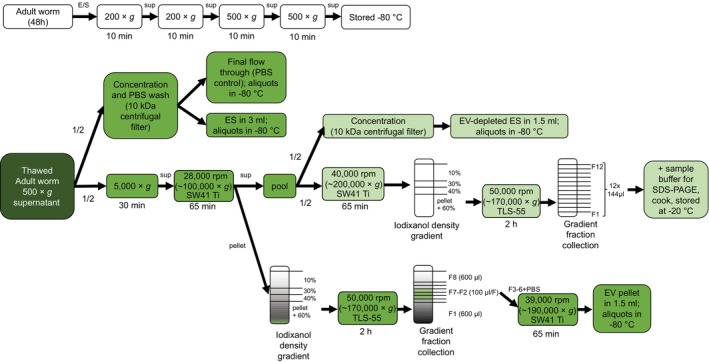
Schematic overview of procedures for preparation of adult worm ES, EV‐depleted ES and purified EVs used for B cell stimulations. E/S, excretory/secretory products; h, hours; min, minutes; rpm, rotations per minute; sup, supernatant.

An overview of the steps for EV isolation from the adult worm ES can be found in Figure 1. Thawed 500 × *g* supernatant from multiple worm cultures (160 mL total) was pooled and equally split in two. One half (80 mL) was concentrated for ES in 15 mL 10 kDa centrifugal filters (Amicon) and washed several times with PBS until the flow‐through was clear. The final ES volume of 3 mL was aliquoted and stored at −80°C. The final flow‐through was also collected, aliquoted and stored to be used later as ‘PBS control’. The other half (80 mL) was processed for EV isolation, which has been described in full detail previously [14]. Briefly, the 500 × *g* supernatant was centrifuged for 30 min at 5000 × *g*, after which the 5000 × *g* supernatant was ultracentrifuged at 100,000 × *g* for 65 min (k‐factor 265, SW41 Ti rotor, Beckman Coulter, Brea, CA, United States). All centrifugation steps were performed at 4°C. The 100,000 × *g* supernatant (‘EV‐depleted ES’) was collected, pooled and split equally into two parts again. One half was concentrated similarly to the ES described above to a volume of 1.5 mL. The other half was transferred to SW41 tubes and centrifuged at 200,000 × *g* (40,000 rpm, k‐factor 130) for 65 min. The 100,000 × *g* pellets were resuspended in a total of 260 μL PBS + 0.2% BSA and divided over two TLS55 tubes (130 μL/tube) and iodixanol (Optiprep, Axis‐Shield PoC AS, Oslo, Norway) gradients were built on top. After 2 h ultracentrifugation at 50,000 rpm (k‐factor 60), 8 fractions were collected from top to bottom: 600 μL (F1), six times 100 μL (F2‐7) and one left over (F8) fraction. Fractions 3–6 were transferred to an SW41 tube and mixed with cold PBS. EVs were pelleted at 39,000 rpm (k‐factor 136) for 65 min and resuspended in 1.5 mL PBS. The final resuspended EV pellet was deliberately half of the ES volume to compensate for the estimated loss of EVs along the density gradient isolation process [25] (Figure S1C). All samples were only thawed once and directly used to stimulate cells. BCA was performed to determine the protein concentration of the ES.

The 200,000 × *g* pellet was resuspended and transferred to a TLS55 tube. An iodixanol gradient was built on top and spun as above. Twelve equal fractions were collected from the gradient, which were directly mixed with non‐reducing sample buffer, cooked and stored at −20°C till SDS‐PAGE and TSP2 western blotting as described in [25].

We have submitted all relevant data of our experiments to the EV‐TRACK knowledgebase (EV‐TRACK ID: EV230956) [26].

### 2.6 Mouse Splenic B Cell Isolation and Stimulation

Spleens of C57/Bl6 mice (SC1300012, purchased by Envigo RMS B.V., Horst, The Netherlands) between 7 and 15 weeks of age were harvested and made into a single cell suspension using a 100 μm cell strainer (BD Biosciences, Franklin Lakes, NJ, United States). B cells were isolated from the splenocytes with CD19 targeting microbeads (Miltenyi Biotec, Bergisch Gladbach, Germany) or CD19 targeting MojoSort nanobeads (BioLegend, San Diego, CA, USA) according to the manufacturer's instructions. Purity was > 96%, which was determined by flow cytometry detecting B220 (Invitrogen, clone RA3‐6B2).

B cells were resuspended in RPMI 1640 glutamax (Gibco) supplemented with 5% FCS (Bodinco BV, Alkmaar, The Netherlands), 100 U/mL penicillin, 100 μg/mL streptomycin and 50 μM 2‐mercaptoethanol (Sigma‐Aldrich). About 500,000 B cells per well (96‐wells plate, U bottom) were stimulated for 2 or 3 days with SEA (20 μg/mL), adult worm ES (12.5, 25, 50, 100 μg/mL), adult worm ES FPLC fractions (50 μg/mL), adult worm EVs (equal volume as adult worm ES), adult worm EV‐depleted ES (equal volume as adult worm ES) or PBS control (ES flow through, equal volume as highest concentration ES), in the presence or absence of co‐stimulatory rat‐α‐mouse CD40 (0.5, 1, 2 μg/mL; clone 1C10, BioLegend) as indicated in the figure legends. After stimulation, the supernatants were collected and stored at −20°C.

### 2.7 Staining of Murine B Cells for Confocal Microscopy and Flow Cytometry

Murine splenic B cells isolated as above were seeded at 375,000 B cells per well (96 well U‐bottom plate) and stimulated with adult worm ES, EVs and PBS control. An equal volume of EV and PBS control was used as calculated for the ES to get an ES concentration of 50 μg/mL. After 18 h incubation, B cells were transferred to a V‐bottom plate and washed twice with PBS. For confocal microscopy, cells were fixed with PFA (3.6%), subsequently permeabilised (eBioscience, Thermo Fischer Scientific with Permeabilisation Buffer, Invitrogen, Thermo Fisher Scientific) and blocked in PBS + 10% FCS for 1 h at room temperature. Cells were incubated overnight at 4°C with or without anti‐TSP2 (1:20,000) (Rabbit IgG, polyclonal, kind gift from Prof. Alex Loukas, James Cook University, Australia). After four PBS washes, cells were incubated for 1 h with an AlexaFluor‐594‐Donkey‐anti‐rabbit IgG (1:500) (Invitrogen) in PBS + 10% FCS. Cells were washed again three times, followed by 30 min incubation with Hoechst (1:200) (33342, Sigma‐Aldrich), one washing step and 30 min CellBrite green (1:100) (Biotium, Fremont, CA, USA). Both Hoechst and CellBrite were diluted in PBS + 2% FCS. After a final wash, cells were transferred to a confocal 96 well plate and spun briefly to sediment the cells. Images were taken using an Andor Dragonfly 500 spinning disk confocal system (Oxford Instruments, Abingdon, UK) with a 60× or 100× objective. For flow cytometry, cells were treated similarly, but including a 20 min Aqua live/dead staining (Invitrogen) before fixation, and after the secondary antibody and washing steps, the cells were resuspended in a buffer for flow cytometry and measured on a BD LSRFortessa (BD Biosciences).

### 2.8 Isolation and Stimulation of Human B Cells

Isolated peripheral blood mononuclear cells (PBMCs) from venous whole blood of healthy volunteers (as described in [27]) were cryopreserved as described [28] till further use. B cells from thawed PBMCs were purified by using a human Pan B Cell Isolation Kit (Miltenyi Biotec) following the manufacturer's protocol. B cell purity was checked by flow cytometry (CD19, clone HIB19, BioLegend) and was > 90%. B cells were seeded in a 96‐well U‐bottom plate, 300,000 cells per well, in RPMI 1640 supplemented with 10% FCS, penicillin and streptomycin as described above and L‐glutamine (2 mM)/pyruvate (1 mM) (Sigma‐Aldrich). After 44 h of stimulation with 50 μg/mL adult worm ES, adult worm EVs, adult worm EV‐depleted supernatant or PBS control (all equal volume as ES), the supernatant was collected and stored at −20°C till cytokine measurements.

### 2.9 Elisa

Culture supernatants from the B cells were thawed and cytokines were quantified by commercial ELISA kits (mouse IL‐6, mouse IL‐10, human IL‐6, human IL‐10; BD OptEIA, BD Biosciences) according to the manufacturer's instructions. Technical duplicates were averaged and statistical analysis was performed on data from three or more independent experiments in GraphPad Prism (version 9, GraphPad Software Inc., La Jolla, CA, United States).

## 3 Results

### 3.1 Adult Worm ES Induces IL‐10 Release by Mouse Splenic B Cells

**FIGURE 2:**
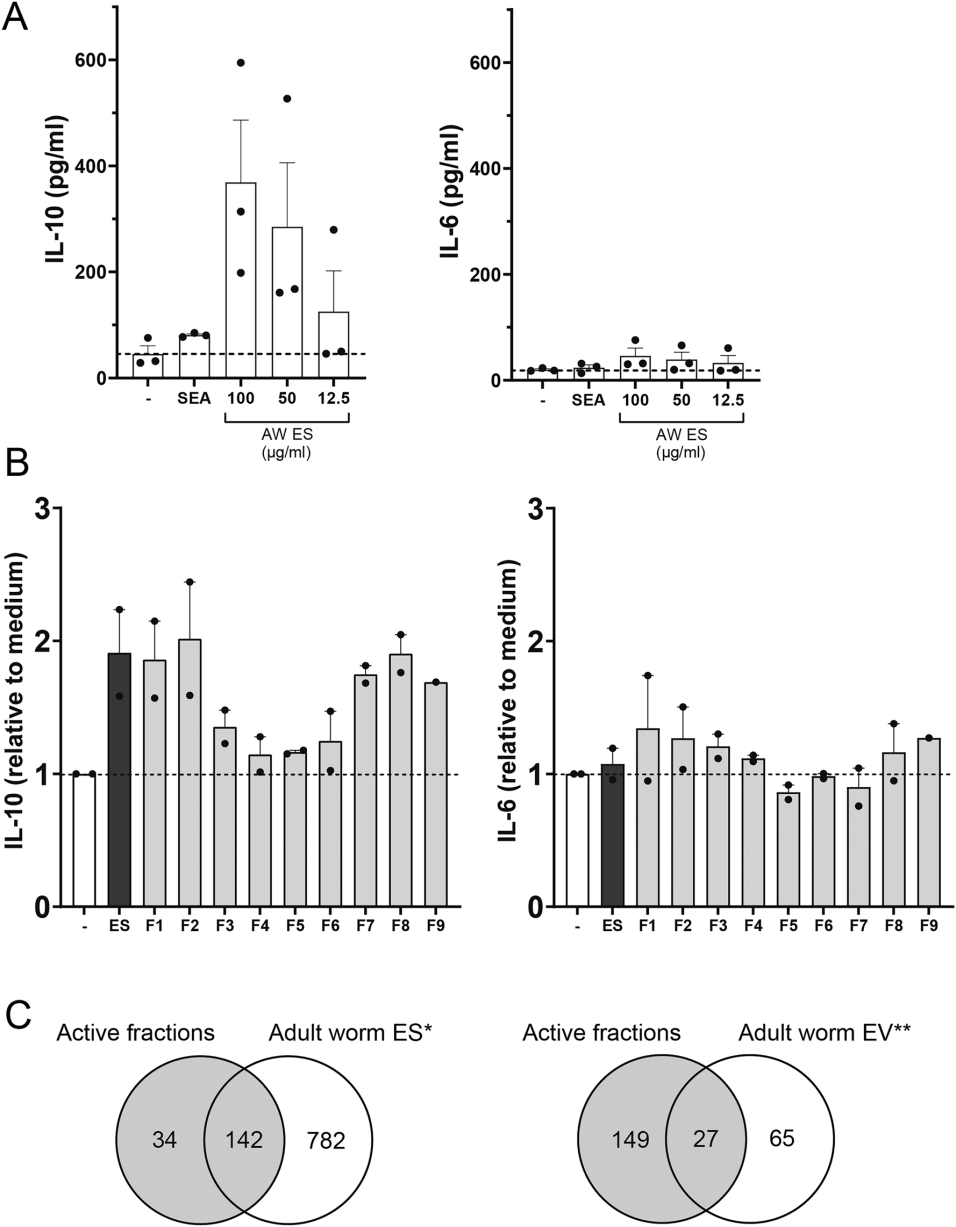
Adult worm ES induces IL‐10 production by murine splenic B cells. (A) IL‐10 and IL‐6 release by 3 day stimulated mouse splenic B cells (*n* = 3). SEA concentration is 20 μg/mL. Error bars indicate SEM. AW ES, adult worm excretory/secretory products; SEA, soluble egg antigen. (B) Similar as in A, with stimulations of 50 μg/mL complete ES or 50 μg/mL of indicated fractions of ES, previously collected by size fractionation. Data of 2 independent experiments. (C) Proteins from proteomic analysis in combined active ES fractions F1 and F7 as compared to published proteins in adult worm ES* [29] or EVs** [9, 11, 12].

We first investigated the effects of adult worm secretions (ES) on the activation of mouse splenic B cells. For this, isolated splenic CD19^+^ cells from a naïve mouse were incubated with either SEA or adult worm ES. Both stimuli induced the release of IL‐10, but not IL‐6, by the B cells and the response by the worm ES was dose dependent (Figure 2A). Next, we questioned whether there are specific molecules present in ES that harbour the IL‐10‐inducing capacity. For this, ES was fractionated by size‐exclusion FPLC (Figure S1B), in which it was expected that similar to other size exclusion chromatography methods [25], EVs would elute in the first fraction after the void volume. Mouse splenic B cells were subsequently incubated with the collected fractions and showed that IL‐10 production was most pronounced after stimulation with fractions 1, 2, 7, 8 and 9 (Figure 2B). These results indicate that in these fractions there is an enrichment of specific ES components that induce IL‐10 release by mouse splenic B cells.

We next investigated whether IL‐10 inducing fractions of adult worm ES contain possible regulatory proteins. Lane and band selection for in‐gel trypsin digestion was guided by signals observed in complementary detection assays that indicated the presence of proteins and/or glycosylated proteins, that is, by Coomassie or ConA (Figure S1B). Based on protein bands in adjacent lanes that either did or did not induce IL‐10 producing B cells, we selected bands from lane 1 and lane 7 as representative fractions for further analysis. The results of the protein identification by mass spectrometry are summarised in the Supporting Information. The identified proteins were compared to the recently published proteome of the complete secretome of cultured male and female adult worms [29] and showed an > 80% overlap in detected proteins (Figure 2C). Among these proteins were regulatory proteins from the 14‐3‐3 family [30] and heat shock proteins (HSP) [31] but also other proteins with immunomodulatory potential such as glyceraldehyde 3‐phosphate dehydrogenase (GAPDH) [32], cathepsin [33], malate dehydrogenase [34] and calmodulin [35]. Interestingly, 14‐3‐3 and HSP are often found in eukaryotic EVs [5]. Two tetraspanins (G4VS95: TSP‐1 [36] and A0A5K4F8N6: TSP‐2 [29]), a family of proteins commonly associated with EVs Automatic citation updates are disabled. To see the bibliography, click Refresh in the Zotero table [7] and known to be present on adult worm EVs [9, 25], were also among the detected proteins. Next, we compared our proteomic data set to proteins that were reported in at least 2 of the 3 current publications on *S. mansoni* adult worm EV protein content [9, 11, 12]. We found that 27 of the 176 (15%) proteins in the active fractions were previously reported to be EV‐associated. This percentage increased to almost 45% when including proteins that have been reported once among the other studies (Table 1). Interestingly, the enrichment of EV‐proteins was highest (40%) in FPLC fraction 1 compared to fraction 7 (21%) (Table 2). This first collected fraction contains large‐sized proteins but also includes molecular complexes and particles, such as EVs. These results suggest that the IL‐10 inducing fractions were enriched for EV‐associated proteins and that EVs among the ES products might influence mouse naïve splenic B cells.

**TABLE 1:** Overview of adult worm ES fraction proteins also reported as EV‐associated in previous studies.

176 proteins detected in active ES fractions	# EV proteins in publications	# of proteins in both EV publications and detected in active worm ES fractions	# proteins only in EV publications	# proteins only found in active ES fractions	% of proteins detected in active ES fractions that are also reported as EV‐associated proteins
EV‐associated proteins reported in ≥ 2 publications on adult worm EV	92	**27**	65	149	**15.3**
EV‐associated proteins reported in ≥ 2 publications on schistosome EV	118	**38**	80	138	**21.6**
EV‐associated proteins reported in ≥ 1 publications on adult worm EV	338	**79**	259	97	**44.9**

*Note:* Comparison of EV‐associated proteins detected in the combined proteomes of F1 and F7 with those published in the literature [8, 9, 11, 12]. The more EV proteins were included, the more overlap with the ES fractions (most right column). Bold values are highlighted for emphasis.

**TABLE 2:** Enrichment of EV‐associated proteins in first collected FPLC fraction of adult worm ES.

	# total detected proteins	# same proteins reported in adult worm EV	% EV‐associated proteins detected in fraction
Fraction 1	37	15	**40.5**
Fraction 7	161	35	**21.7**

*Note:* Higher percentage of adult worm EV‐associated proteins detected in Fraction 1 from adult worm ES (most right column). Adult worm EV proteins included were reported in at least two adult worm EV publications [9, 11, 12]. Bold values are highlighted for emphasis.

### 3.2 Adult Worm EVs Interact With Mouse B Cells

**FIGURE 3:**
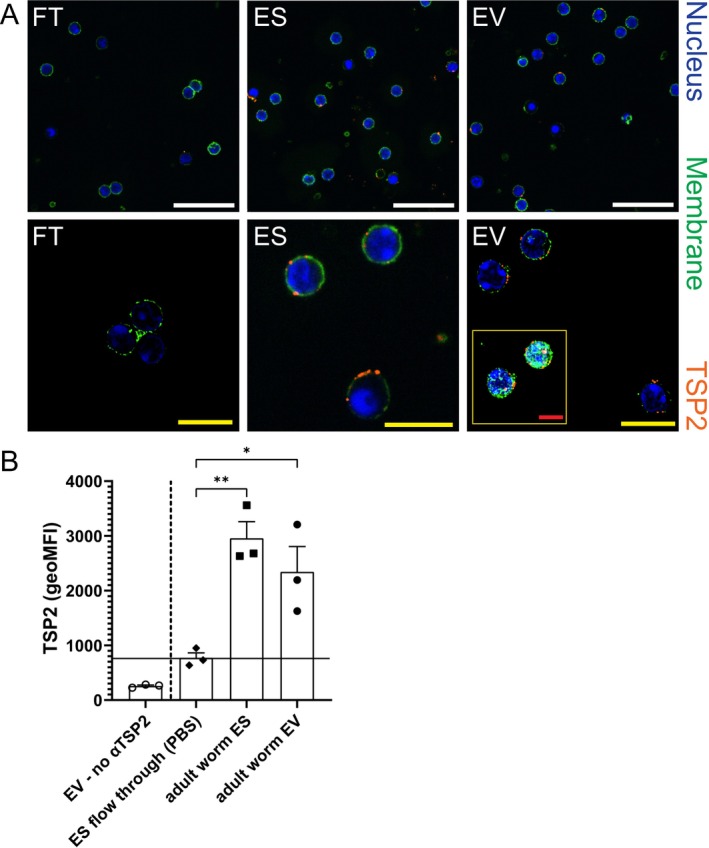
*Schistosoma* adult worm EVs interact with mouse B cells. Mouse splenic B cells were incubated with ES flow through (FT, equal to PBS), worm ES, or worm EVs, all to the same amount as 50 μg/mL ES, for 18 h. Presence of schistosome EVs is shown by a *Schistosoma*‐specific TSP2 targeting antibody. (A) Confocal microscopy shows the presence of EV marker TSP2 (anti‐TSP2, orange) around the membranes (Cellbrite, green) of the B cells (Hoechst, nuclei are blue) after ES and EV incubation. Scale bars: white = 30 μm; yellow = 10 μm; red = 5 μm. Pictures are either a section of the cells or z‐stack projections (insert). (B) Average TSP2 geometric mean fluorescent intensity (geoMFI) of the incubated B cells measured by flow cytometry. The signal in PBS stimulated B cells represents unspecific binding of the TSP2 antibody. Cells incubated with EVs but no TSP2 antibody excluded binding from the secondary antibody alone. Data from three independent experiments. Error bars indicate SEM. One‐way ANOVA with Dunnett's multiple comparisons test; *p < 0.05, **p < 0.01.

To investigate whether adult worm EVs indeed interact with B cells, complete adult worm ES and purified adult worm EVs from this ES were incubated with mouse splenic B cells which were subsequently stained with an *S. mansoni* tetraspanin‐2 (TSP2)‐specific antibody and analysed by fluorescence microscopy and by flow cytometry. Detailed characterisation of similar isolated adult worm EVs by nanoparticle tracking analysis (NTA) and cryo‐electron microscopy has been reported in our previous publications and is further detailed in EV‐TRACK [14, 25]. B cells incubated with ES and with EVs showed the presence of TSP2 at the plasma membrane (Figure 3), suggesting that both purified EVs and EVs in total adult worm ES interacted with the cells. Interaction of EVs with the B cells seemed unaffected by pre‐incubating the cells with EGTA or pre‐incubating the EVs with the TSP2 targeting antibody (Figure S2). From these data, we can conclude that the EVs in ES released by adult worms interact with mouse B cells in vitro.

### 3.3 EVs Released by Adult Worms Increase IL‐10 Production by Mouse B Cells

**FIGURE 4:**
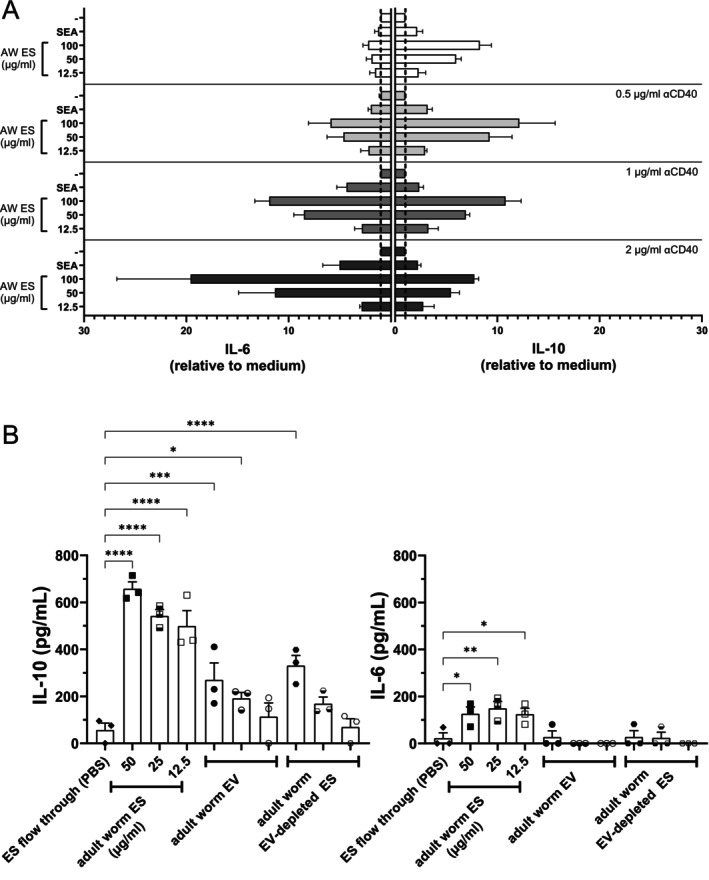
Adult worm EVs induce IL‐10 production by murine splenic B cells. (A) Cytokine production of mouse splenic B cells stimulated with ES for 3 days increased with additional CD40 stimulation (*n* = 3). SEA concentration is 20 μg/mL. Error bars indicate SEM. (B) Cytokine release by mouse splenic B cells stimulated with ES, EVs of EV‐depleted ES for 2 days. The volumes of resuspended purified EVs and EV‐depleted ES added were equal to the ES. All conditions were in the presence of 0.5 μg/mL αCD40. Data points are averaged technical duplicates from three independent experiments. Error bars indicate SEM. One‐way ANOVA with Dunnett's multiple comparisons test *p < 0.05, **p < 0.01, ***p < 0.001, ****p < 0.0001.

Since it was previously shown that additional CD40 ligation enhanced IL‐10 release by mouse splenic B cells in response to SEA [20], we tested whether the same applies to adult worm ES. Indeed, CD40 co‐stimulation increased cytokine responses in response to adult worm ES (Figure 4A). Since a dose of 0.5 μg/mL of the CD40 co‐stimulatory antibody (as compared to 1 and 2 μg/mL) resulted in the highest IL‐10 release with minimal increase in IL‐6 production (Figure 4A), we continued with this dose αCD40 in following experiments.

To study the contribution of EVs within the adult worm ES on B cell responses, equal volumes of worm ES, EVs purified from ES, and EV‐depleted ES were added to naïve splenic B cells. After 48 h, both purified EVs and the EV‐depleted ES increased IL‐10 release by stimulated B cells, but to a lower extent than total adult worm ES (Figure 4B). This may suggest that IL‐10 inducing signals are present both within EVs and non‐EV components. However, we observed that a residual amount of EV‐associated TSP2 protein could still be purified from the ‘EV‐depleted’ adult worm ES fraction (Figure S1D). Thus, it cannot be excluded that contaminating EVs in the EV‐depleted adult worm ES influenced the cytokine production by B cells exposed to this material. In contrast to complete adult worm ES, the EVs and EV‐depleted ES did not induce IL‐6 release by mouse splenic B cells. This unexpected result may indicate that IL‐6‐inducing components act synergistically in the complete ES, or that critical factors were at too low a concentration due to loss during sample preparation. Combined, these results suggest that adult worm EVs can induce IL‐10 release by mouse B cells.

### 3.4 Adult Worm EVs Stimulate Cytokine Responses by Human B Cells

**FIGURE 5:**
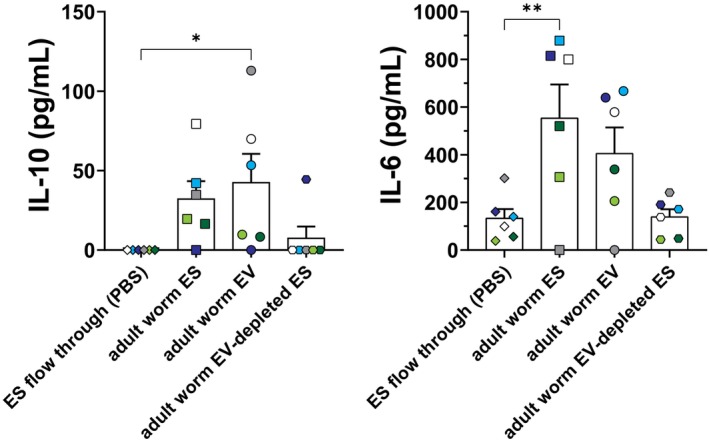
Cytokine production of adult worm ES and EVs stimulated human B cells. IL‐10 and IL‐6 release by human B cells isolated from peripheral blood of 6 healthy individuals after 2 days of stimulation with adult worm ES, EVs, or EV‐depleted ES. Equal volume of EV and EV‐depleted ES was used as for the 50 μg/mL ES stimulation. Each individual donor corresponds to one and the same colour. Error bars indicate SEM. One‐way ANOVA with Dunnett's multiple comparisons test *p < 0.05, **p < 0.01.

Finally, we questioned whether adult worm ES and EVs could also activate human peripheral blood B cells. B cells were isolated from PBMCs from six healthy individuals and exposed to complete adult worm ES, adult worm EVs or EV‐depleted ES. Complete ES and purified EVs both induced an increase in IL‐10 and IL‐6 release, while the EV‐depleted ES did not seem to induce (strong) cytokine responses (Figure 5). Not all donors showed the same responses to the adult worm released products, which points at some heterogeneity in the responses between donors. Nevertheless, these data indicate that adult worm ES and their purified EVs can induce both IL‐6 and IL‐10 responses in human peripheral blood B cells.

## 4 Discussion

Schistosome secretions modulate host immune responses to enable parasite survival [5], in part through the activation of immune tolerance and increased IL‐10 producing B cells [18]. Here we showed that schistosome adult worm ES induces IL‐10 release by both murine splenic B cells and human peripheral B cells. Both adult worm EVs as well as non‐EV‐associated factors seem to harbour this activity.

The finding that highly purified adult worm EVs can modulate B cell immune responses contributes to a limited number of studies on immunoregulatory effects of (mammalian) EVs on B cells [37, 38, 39]. For example, EVs from mesenchymal stromal/stem cells (MSC) can inhibit B cell proliferation and activation via miRNA transfer [40], while mast cell‐derived EVs increased IL‐10 competent murine B cells via EV‐associated CD40L [41] or attenuated pro‐inflammatory cytokine expression by B cells via prostaglandins [37]. While it seems unlikely that worm‐derived EVs contain (host‐derived) CD40L, prostaglandins have been found in adult worm ES [42]. However, it is unknown whether prostaglandins are also present in worm EVs.

Proteomic analysis of the IL‐10 inducing fractions (Supporting Information) revealed the presence of EV‐associated proteins such as Sm29, Sm22.6, tetraspanins, 14‐3‐3 protein, L‐lactate dehydrogenase and enolase [9, 12]. Recombinant versions of Sm29, Sm22.6, and tetraspanin (namely TSP2) have been tested as potential vaccine candidates against schistosomes, of which Sm29 and TSP2 showed reduced egg and worm burden after immunisation [43, 44]. Interestingly, Sm29 and Sm22.6 also seem to have regulatory functions: for example, Sm29 increased IL‐10 production in human monocyte‐derived dendritic cells [45] and both Sm29 and Sm22.6 increased IL‐10 production in murine splenic cells [43, 46]. The mammalian 14‐3‐3 protein was able to enhance B cell survival, proliferation and expansion [47], while its 14‐3‐3ε isoform has anti‐inflammatory properties as part of the TNF receptor‐associated factor 2 [48]. The 14‐3‐3ε protein of *S. mansoni* can bind to human type 1 TGFβ receptor [49]. Intriguingly, targeting mammalian TGFβ receptors to stimulate tolerance was demonstrated for Treg cell inducing protein *Hp*‐TGM released by *Heligmosomoides polygyrus* [50]. The TGFβ receptor is also present on B cells [51], but whether *S. mansoni* or *H. polygyrus* induces IL‐10 release from B cells via this route is currently unknown.

Enolase and L‐lactate dehydrogenase (LDH) are both important in the cell metabolism pathway towards lactate production. Schistosomes depend on glucose metabolism for their survival, which also results in increased lactate levels [52]. Lactate as an immunomodulator is well known within the tumour field, as for example, Tregs thrive in a lactate‐rich environment and their proliferation and immunosuppressive capacity highly depend on lactate consumption as a fuel source [53]. Invasive tumours are known to increase LDH, which has subsequential immunosuppressive effects [54]. Interestingly, there seems to be a role for B cell‐expressed LDH in the expansion of IL‐10 producing Breg cells [55]. Furthermore, lactate can bind to G‐protein coupled receptor (GPR)65 (TDAG8) that is present on human and mouse B cells, and was shown to reduce pro‐inflammatory cytokine production, such as IL‐6, in mouse bone marrow‐derived macrophages [56]. Whether the EV‐associated adult worm ES proteins we detected by mass spectrometry are actually taken up by B cells and contribute to IL‐10 induction is unknown.

A possible component with immunomodulatory potential in adult worm ES is hemozoin [57]. This crystal form of the otherwise toxic blood digestion product heme was taken up by B cells, as shown for hemozoin from *Plasmodium falciparum*, and was stimulating TLR9 in B cells via its bound malarial DNA [58, 59]. It is possible that part of the immune responses induced by the worm ES is due to its hemozoin. However, we have used an optimised EV isolation protocol for adult worm EVs that excludes hemozoin from the isolated EVs [25], so we argue that the EV‐mediated IL‐10 B cell responses are hemozoin‐independent.

Even though we do not know which individual EV molecule(s) stimulate(s) B cell IL‐10 production, we provide strong evidence that the adult worm EVs interact with B cells (Figure 3). We used schistosome specific TSP2‐targeting antibodies to detect EV‐B cell interaction. However, this detection does not reveal whether it was the whole EV population or a subpopulation that interacted with the B cells, since information on TPS2 expression on EV‐subsets from schistosomes is still lacking. It is known for mammalian EVs that the tetraspanin profile on EVs can differ per in vitro or in vivo source [60]. This also raises the question of which of the isolated adult worm EVs were released by males or females in our mixed worm cultures, as both sexes have a different protein expression profile with potentially different immunomodulatory consequences [29, 61]. IL‐10 producing B cells have been identified in male only infections and in mixed‐sex infections [19, 22], but the effect of female only secretions on immune cells has not been reported. Whether the EVs remain present at the B cell surface or are internalised by the cells or fuse with the membrane is something we could not evaluate by our TSP2 detection assay. Studying the fate of the worm EVs after interaction with the B cells will also provide directions for which molecules (surface or cargo) are most likely involved in the immune modulation.

The adult worm EVs may interact with B cells via a receptor or a receptor‐independent mechanism. We know that these EVs are highly glycosylated [14] and that schistosome EVs can bind to antigen‐presenting cells via EV‐associated glycans [14, 15]. However, initial tests of pre‐treating the B cells with EGTA to block glycan‐lectin interactions or pre‐treating the adult worm EV with the αTSP2 antibody did not reduce the interaction of the EVs with the B cells (Figure S2). This suggests that C‐type lectin receptors and TSP2 on the EVs are not essential for EV‐B cell interaction and the putative downstream IL‐10 induction. B cells express only a few CLRs compared to myeloid cells, and the B cell CLRs seem to have a bigger role in self‐recognition and homeostasis [62] and only a little is reported on their role in pathogen recognition [63]. However, since anti‐glycan antibodies targeting schistosome glycans have been reported in naïve individuals [64], there is a possibility that adult worm EVs and their associated glycans interact with B cell receptors recognising those glycans. Future studies should address whether this putative interaction exists.

The differences in IL‐6 response between the mouse (Figure 4) and human B cells (Figure 5) might be attributed to the fact that the B cell species and sources are different (spleen versus peripheral blood) and may represent different populations. For example, unstimulated human B cells lack TLR3 and TLR4 expression while mouse splenic B cells do express those receptors [65]. Furthermore, human peripheral blood B cells produce both IL‐10 and IL‐6 in response to various TLR stimulations, with or without additional CD40 ligation or added cytokines, while those B cell populations still exerted immune suppressive effects [66]. However, it is unclear to which extent IL‐10 producing human peripheral blood and mouse splenic B cells induced by adult worm EVs have similar regulatory capacities.

This study shows that *Schistosoma*‐derived EVs can target and affect B cells. This emphasises that these blood‐residing parasites have developed diverse and complex survival mechanisms via host immune modulation in which schistosome EV‐host cell interactions should be considered as an important factor. *Schistosoma* EVs should therefore be considered in the design of novel vaccination strategies and/or strategies to target hyperinflammatory disorders.

## Author Contributions

A.O.‐F. and M.E.K. performed the majority of the experiments. S.H. contributed to the ES isolation and B cell stimulations. S.H., N.N.D. and P.J.H. performed the proteomics experiments. L.M. compared the proteome data to the literature. C.M.K. generated the microscopy images. H.H.S., S.H. and M.E.K. conceived the project. M.E.K., A.O.‐F., H.H.S., E.N.M.N.H. and C.H.H. contributed to the design of the experiments and the interpretation of the results. M.E.K., A.O.‐F. and H.H.S. wrote the manuscript. All authors revised the manuscript. All authors read and approved the final manuscript.

## Ethics Statement

Animal experiments in this manuscript were approved by the Leiden University Medical Center Ethical Review Board and registered under AVD1160020171067 and AVD1160020173525.

## Conflicts of Interest

The authors declare no conflicts of interest.

## Peer Review

The peer review history for this article is available at https://www.webofscience.com/api/gateway/wos/peer‐review/10.1111/pim.70023.

## Supporting information


**Data S1:** pim70023‐sup‐0001‐DataS1.xlsx.

**Figure S1:** Representative chromatogram fractions of adult worm ES and adult worm EV isolation by density gradients.
**Figure S2:** No effect of pre‐incubation of EVs with αTSP2 or pre‐incubation of cells with EGTA on EV‐B cell interaction.

## Data Availability

This manuscript contains data that is shared via the PRIDE partner of the ProteomeXchange Consortium repository (PXD044023).
