# Supplementary material for: Extracellular Vesicles From Schistosoma mansoni Adult Worms Stimulate IL‐10 Release by B Cells
Source: Parasite Immunol. 2025 Sep 7;47(9):e70023. doi: 10.1111/pim.70023 (PMC12415506; doi:10.1111/pim.70023)
Supplement: Supplementary file 2 — Figure S1: Representative chromatogram fractions of adult worm ES and adult worm EV isolation by density gradients. Figure S2: No effect of pre‐incubation of EVs with αTSP2 or pre‐incubation of cells with EGTA on EV‐B cell interaction. [file PIM-47-e70023-s001.docx]

**Supplementary figures corresponding to “Extracellular vesicles from *Schistosoma mansoni* adult worms stimulate IL-10 release by B-cells”**


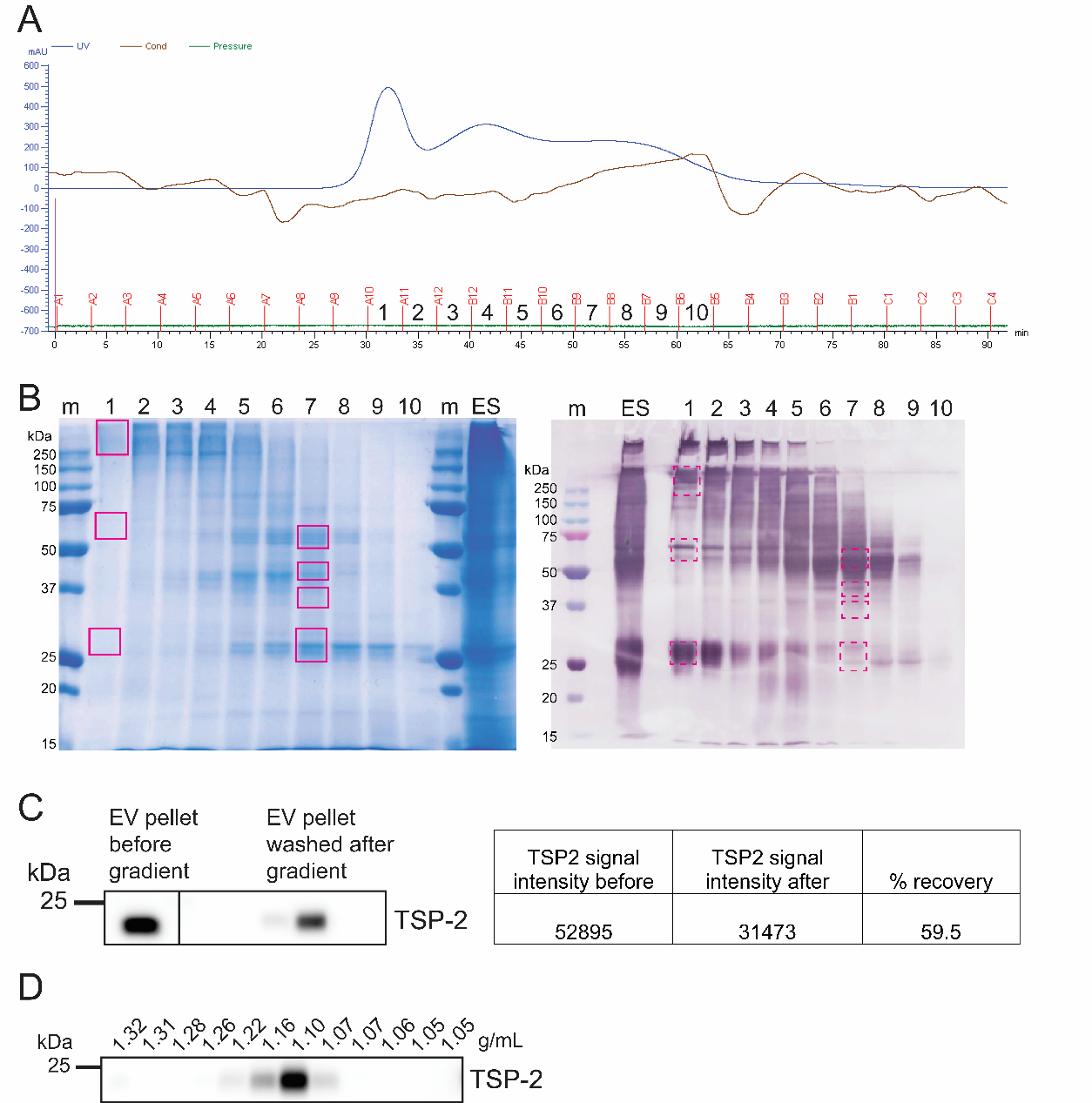


**Supplement Figure S1. Representative chromatogram fractions of adult worm ES and adult worm EV isolation by density gradients**

**A**: Representative gel filtration chromatograph of one run of complete adult worm ES on a Sephacryl S-300 HR column. Collected fractions are indicated as 1-10.

**B:** SDS-PAGE coomassie staining of chromatogram fractions 1-10 and total ES (left). Pink squares indicate the areas that were cut for in-gel-digestion and proteomics analysis. Immunoblot for Concanavalin A (ConA) showing (glycosylated) proteins in each ES fraction (right). Dashed pink squares indicate the overlap with the cut gels from the coomassie staining. m, marker

**C:** EVs in a 100,000 ×*g* pellet were equally split and one half was subjected to an iodixanol density gradient purification step after which the EV containing fractions were pooled and EVs re-pelleted and resuspended in the same volume as the other half. EVs before and after the gradient were checked for the marker TSP2 by western blotting and signals intensities were determined with ImageJ. Loss of EVs is >40%.

**D**: The 100,000 ×*g* EV-depleted ES was ultracentrifuged at 200,000 ×*g* and the pelleted material was subjected to iodixanol density gradient centrifugation, after which 12 density fractions were collected. Western blot of TSP2 shows that adult worm EVs are still present in the EV-depleted ES.


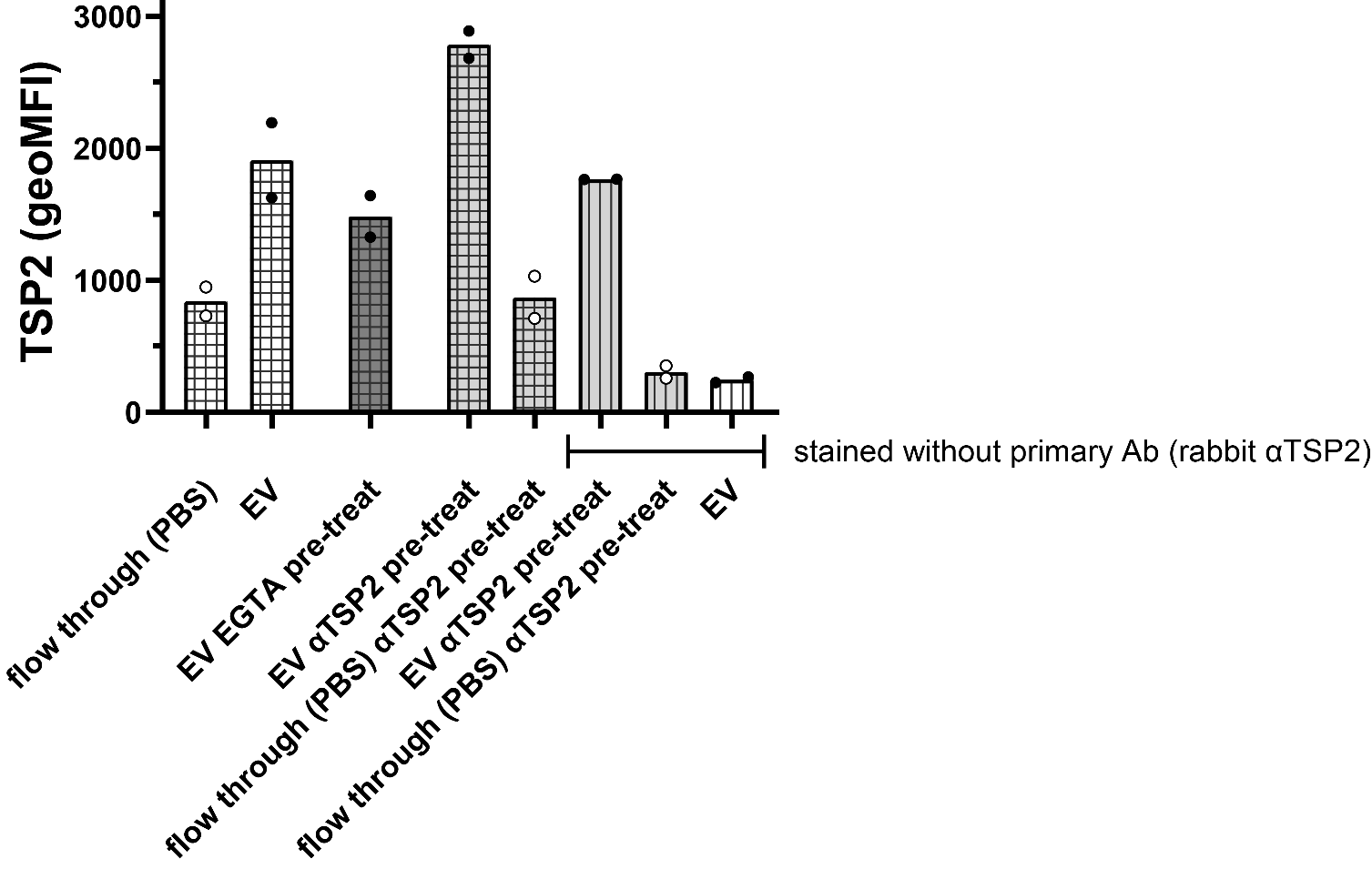


**Supplement Figure S2. No effect of pre-incubation of EVs with αTSP2 or pre-incubation of cells with EGTA on EV-B cell interaction**

EVs and ES flow through (FT, equal to PBS) were or were not pre-incubated for 30 minutes with αTSP2 (1:500) (αTSP2 pre-treat). Splenic B cells from mice were pre-incubated with or without EGTA (10 mM) for 30 minutes before incubation with adult worm EVs (EGTA pre-treat). EVs and ES flow through including their pre-treatments were added in similar amount as 50 µg/ml ES and incubated with the B cells for 18 hours. Cells were washed and stained for TSP2 (primary rabbit-αTSP2) and a secondary antibody with fluorochrome (donkey αrabbit) to indicate EV binding. Average TSP2 geometric mean fluorescent intensity (geoMFI) of the incubated B cells was measured by flow cytometry. Data from 2 independent experiments.
